# Antitumor effects of arsenic disulfide on the viability, migratory ability, apoptosis and autophagy of breast cancer cells

**DOI:** 10.3892/or.2020.7653

**Published:** 2020-06-19

**Authors:** Yuxue Zhao, Kenji Onda, Kentaro Sugiyama, Bo Yuan, Sachiko Tanaka, Norio Takagi, Toshihiko Hirano

Oncol Rep 41: 27-42, 2019; DOI: 10.3892/or.2018.6780

Following the publication of the above paper, an interested reader drew to our attention the fact that the same β-actin bands had been included in the western blots featured in Figs. 7 and 10. Upon consulting the authors in relation to this matter, they were able to offer a legitimate explanation for this apparent duplication of the bands; essentially, in the western blotting experiments, different antibodies were used in one membrane to present different target proteins by stripping and reprobing the membrane. That the authors had performed this additional step in their protocol was inadvertently omitted from the ‘Western blot analyses’ subsection of the Materials and methods section.

The authors are grateful to the interested reader for drawing this matter to their attention, and apologize for any inconvenience caused to the readership of the Journal.

